# Functional Trait Trade-Offs for the Tropical Montane Rain Forest Species Responding to Light from Simulating Experiments

**DOI:** 10.1155/2014/649031

**Published:** 2014-06-11

**Authors:** Peili Mao, Runguo Zang, Hongbo Shao, Junbao Yu

**Affiliations:** ^1^Key Laboratory of Coastal Zone Environmental Processes, Yantai Institute of Coastal Zone Research, Chinese Academy of Sciences (CAS), Yantai, Shandong 264003, China; ^2^Key Laboratory of Forest Ecology and Environment, The State Forestry Administration, Research Institute of Forest Ecology, Environment and Protection, Chinese Academy of Forestry, Beijing 100091, China; ^3^Institute of Life Sciences, Qingdao University of Science & Technology, Qingdao 266042, China

## Abstract

Differences among tropical tree species in survival and growth to light play a key role in plant competition and community composition. Two canopy species with contrasting functional traits dominating early and late successional stages, respectively, in a tropical montane rain forest of Hainan Island, China, were selected in a pot experiment under 4 levels of light intensity (full, 50%, 30%, and 10%) in order to explore the adaptive strategies of tropical trees to light conditions. Under each light intensity level, the pioneer species, *Endospermum chinense* (Euphorbiaceae), had higher relative growth rate (RGR), stem mass ratio (SMR), specific leaf area (SLA), and morphological plasticity while the shade tolerant climax species, *Parakmeria lotungensis* (Magnoliaceae), had higher root mass ratio (RMR) and leaf mass ratio (LMR). RGR of both species was positively related to SMR and SLA under each light level but was negatively correlated with RMR under lower light (30% and 10% full light). The climax species increased its survival by a conservative resource use strategy through increasing leaf defense and root biomass investment at the expense of growth rate in low light. In contrast, the pioneer increased its growth by an exploitative resource use strategy through increasing leaf photosynthetic capacity and stem biomass investment at the expense of survival under low light. There was a trade-off between growth and survival for species under different light conditions. Our study suggests that tree species in the tropical rainforest adopt different strategies in stands of different successional stages. Species in the earlier successional stages have functional traits more advantageous to grow faster in the high light conditions, whereas species in the late successional stages have traits more favorable to survive in the low light conditions.

## 1. Introduction 

In tropical rain forests, light is the most important limiting resource for tree seedling establishment, growth, and survival [1–5]. Shade tolerance plays a major role in plant community dynamics [5–10]. Two important hypotheses for species' shade tolerance have been proposed: carbon gain hypothesis [11] and stress tolerance hypothesis [12]. Carbon gain hypothesis defines shade tolerance as the maximization of net carbon gain together with the minimization of respiration costs for maintenance. However, carbon gain hypothesis has been challenged by some studies on tropical tree seedlings that have not found growth ranking reversals of different shade tolerance species in high and low light [12–14]. Stress tolerance hypothesis thought of shade tolerance as maximization of the resistance to biotic and abiotic stresses in low light. Some new researches agreed with this hypothesis in recent years [13–17].

It has reached a consensus on the suites of traits that associate with shade tolerance, including leaf physiology and biochemistry, leaf anatomy and morphology, crown size, and whole plant architecture [10]. Pioneer species demonstrate higher metabolic rate, such as higher RGR, photosynthesis capacity, and respiration rate, than shade tolerant species [18–22], which is disadvantageous for them to survive in the understory because their metabolic cost of maintaining high photosynthetic performance cannot be supported in low light environment [18]. Small seedlings of pioneer species allocate higher biomass to leaves in order to escape understory constraint by rapid growth [12, 23–25]. On the contrary, shade tolerant species show higher root biomass allocation, which is advantageous for them to escape carbohydrate loss and increase their resprouting ability in the understory [26]. Specific leaf area is an important trait for plant shade tolerance, because it has close relationships with leaf photosynthetic rate, leaf span, and leaf defensive ability [12, 23, 27, 28]. However, some new studies suggested that wood density was a better trait indicating plant shade tolerance [26, 29].

Phenotypic plasticity is an important means for plants to cope with environmental heterogeneity [30, 31]. It was a striking trait associated with shade tolerance [10]. Plant plastic phenotypic responses can enhance light capture and photosynthetic efficiency in the shade [32]. In low light, plant species with higher plasticity show higher seedling mortality rate [33, 34]. Morphological plasticity is an important feature of plants in natural communities. Some researchers reported that pioneer species had higher plasticity in morphology [22, 25, 35]. However, some other studies suggested the contrary conclusions [10, 36–38]. Coste et al. [39] indicated that plasticity of leaf traits between pioneer and shade tolerant species was similar. Rozendaal et al. [40] thought that pioneer species had higher plasticity in seedling stage not at adult stage. So, the relationship between plant plasticity and shade tolerance is complex.

The tropical rain forest on Hainan Island of China is one of the most important forest ecosystems, which has complex structure and rich biodiversity. A large percentage of tropical forests in China are tropical montane rainforests, especially on Hainan Island, which is in the most southern part of China and in the northern edge of tropical Asia [41]. However, the excessive deforestation and unreasonable land use have led to the large scale reduction of primary forest and formation of degraded ecosystems of different successional stages. The natural secondary forest, developing after the primary forest was destroyed, has become the most important forest resources in Hainan Island. There have been many studies on the composition, structure, and dynamics on the tropical montane rain forest of Hainan Island, but few studies have been carried out on the ecophysiology of trees in the forest, which is the basis to further explore the function and dynamics of the forest ecosystems. Studies on the ecophysiology of tree seedlings in different successional stages could help us to understand the mechanism of tree replacement and the relationships between trees and their environment.

In this paper, seedlings of two representative tree species (*Endospermum chinense* (Ec) and* Parakmeria lotungensis* (Pl)) distributed in the tropical montane rain forest on Hainan Island were selected and their ecological adaptation to light was studied. We measured some of the growth and morphological traits of the two species for seedlings under different light levels by the pot experiments. The objectives of the study were to explore the following questions: (1) whether light intensity had any significant influence on the performance of tree species in terms of growth and morphological traits, (2) whether the ecological adaptation strategies to light for the two tree species differed significantly, (3) whether irradiance-elicited morphological plasticity differed among the two species, and (4) whether there were any trade-offs between growth and survival related traits under different light conditions.

## 2. Materials and Methods 

### 2.1. Study Area

The study was carried out in the Jianfengling forest region of Hainan Island, and our pot experiments were conducted at the Jianfengling Experimental Center of The Research Institute of Tropical Forestry, Chinese Academy of Forestry (latitude 18°42′N; longitude 108°49′E; altitude 80 m). The tropical montane rain forest in Jianfengling is distributed from 700 to 1100 m in altitude, which possesses the largest area and the richest species in the Jianfengling forest region. The tropical montane rain forest area in Jianfengling has a mean annual air temperature of 19.7°C. The rainfall averages 2651.3 mm per year. Mean annual relative humidity is 88%. The forest is mainly composed of tree species from the families of Lauraceae, Rubiaceae, Fagaceae, and Myrtaceae.

### 2.2. Study Species

Two evergreen canopy tree species of the tropical montane rain forest were selected due to the limitation of seeds and seedlings. According to the experiences of local foresters and our observations in the forest region,* E. chinense* (Ec, Euphorbiaceae) was regarded as the representative species of pioneer or early successional stage, which was common in the natural secondary forest stands. It is a light demanding pioneer species dominating the early successional stage of the tropical montane rainforest and can be found frequently in the large canopy openings and disturbed areas. Its maximum height is above 30 m. However,* P. lotungensis *(Magnoliaceae) is a shade tolerant climax species dominating the old growth stands of the tropical montane rainforest. It is distributed in fertile broad-leaved forest. It can regenerate well under closed canopy. Its maximum height also is above 30 m. Ec is a fast growing species, which plays important roles in the carbon sequestration of secondary forest; meanwhile, its timber can be used for firewood and utensil makings. Pl is a slower growing hardwood with higher wood density, which has high quality timber for industrial and civil use. Ecologically, the dominant role played by Pl in later successional stages of the tropical montane rainforest makes it a paramount species in carbon storage and functioning of the forest ecosystems. These two species are important species both ecologically and economically in this tropical forest area, which is one of the reasons why we select them for this experimental study. The classification of the tree species into early versus late successional groups was only based on experiences and observations but had no ecophysiological basis, which is just one of the objectives of this experimental study.

### 2.3. Experimental Design

Plants were grown at four different light levels. Three shade chambers were created by cement poles. Three light levels were created by covering the shade chambers with an increasing number of layers of neutral shade netting [42]. The daily course of incident photon flux density (PFD) was measured with an open portable photosynthesis system (LI-6400; Li-Cor, Inc., Lincoln, NE) in March 2005. PFD was measured every two hours from 10:00 to 18:00 o'clock in three cloudless days. The four light levels were about 100%, 50%, 30%, and 10% full light.

Mature seeds of the study species were collected from numerous mother plants in the tropical montane rain forest of Jianfengling Nature Reserve. After seeds were germinated, seedlings were grown in 30% full light in the nursery near the Jianfengling Experimental Center for 8 months. Fifty healthy trees of each species were selected and transplanted to 9.1 L white plastic pots (one seedling per pot) on March 30, 2005. Then, these seedlings were placed under 30% full light to revive for about 50 d until they grew new leaves. After this, twelve individuals per species were moved to each light treatment on May 20, 2005 [2]. All seedlings were watered well. Positions of the pots were designed regularly. No seedlings died during the experiment.

At the time of moving, a sample from the seedlings (*N* = 5 per species) was harvested to measure initial patterns of biomass partitioning. Seedlings were separated into leaves, stems, and roots. Leaf area was measured with a leaf area meter (LI-3000A, Li-Cor, Inc., USA) and the material was oven dried at 70°C to constant weight and weighed to the nearest 0.01 g. Regression equations based on leaf area and leaf maximum width of the initial harvested seedlings were used to estimate the leaf area of unharvested seedlings.

After the experiment ended on March 3, 2006, all seedlings were harvested and each was separated into leaf, stem, and root components. Leaf area was calculated by regression equations, and seedlings were dried at 70°C to constant weight and weighed to the nearest 0.01 g. Seedling height and leaf maximum width were recorded at the start and end of the experiment. Branch number of each seedling was counted at the end of the experiment. From the harvest data, the morphological and growth indexes were derived (Table 1). The plastic index was calculated according to Valladares et al. [35] by the following formula: Plastic index (of a feature) = (maximum − minimum)/maximum.

### 2.4. Data Analysis

Two-way analysis of variance (ANOVA) was used to analyze the effects of light, species, and their interaction on each of the dependent variables. The least significant difference (LSD) multiple comparisons were performed to permit separation of effect means at significant level of *P* < 0.05. Correlation analyses were used to investigate relationships between RGR and other variables under each light level. All statistics were carried out using the SPSS for Windows 13.0 (SPSS, Chicago, IL, USA).

## 3. Results 

### 3.1. Growth Rate

**Figure 1:**
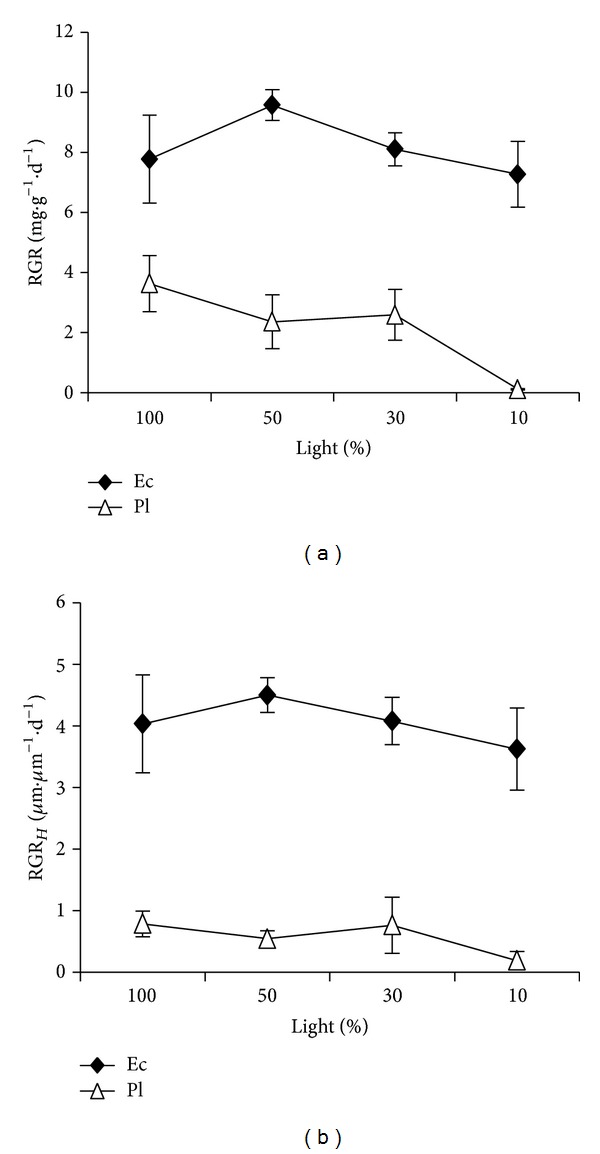
Growth rate responses to light for two rain forest tree species. Figures refer to (a) relative growth rate and (b) relative height growth rate.

Light intensity showed significant effects on relative growth rate (RGR; *F* = 9.08; *P* < 0.01; Figure 1(a)). RGR decreased with the decrease of light intensity. RGR in 100% was similar to that in 50% full light (*P* > 0.05), and they were significantly higher than that in 10% full light (*P* < 0.01). However, light intensity showed no significant effects on relative height growth rate (RGR_*H*_; *F* = 2.51; *P* = 0.08; Figure 1(b)). There were significant differences in RGR (*F* = 327.98, *P* < 0.01) and RGR_*H*_ (*F* = 420.24, *P* < 0.01) between the species. Ec showed higher RGR (*P* < 0.01) and RGR_*H*_ (*P* < 0.01). In 10% full light, RGR and RGR_*H*_ for Pl were 0.11 ± 0.02 mg·g^−1^·d^−1^ and 0.19 ± 0.15 *μ*m·*μ*m^−1^·d^−1^, respectively, which suggested that the light-compensation point for Pl to grow was about 10% of full light. The interaction between light and species was significant for RGR (*F* = 5.10, *P* < 0.01) but not for RGR_*H*_ (*F* = 0.91, *P* = 0.45). With decreasing light intensity, RGR for Ec decreased more slowly than Pl.

### 3.2. Biomass Allocation

**Figure 2:**
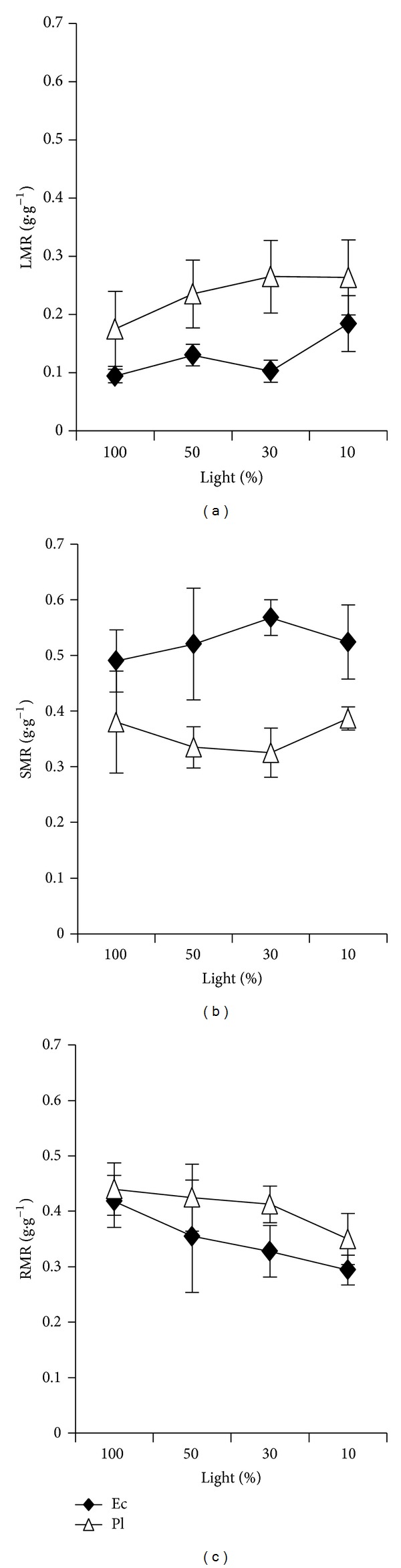
Biomass allocation responses to light for two rain forest tree species. Figures refer to (a) leaf mass ratio, (b) stem mass ratio, and (c) root mass ratio.

Among the light levels, leaf mass rate (LMR; *F* = 5.04; *P* < 0.01) and root mass rate (RMR; *F* = 5.43; *P* < 0.01) differed significantly, whereas stem mass rate (SMR) did not (*F* = 0.30, *P* = 0.83). As the light intensity decreased, the two species increased LMR and decreased RMR, while their SMR did not vary obviously (Figure 2). The results showed that investment was made in stem biomass in low light at the cost of investment in root biomass. The two species differed significantly in LMR (*F* = 42.51, *P* < 0.01), SMR (*F* = 57.71, *P* < 0.01), and RMR (*F* = 9.25, *P* < 0.01). Compared with Pl, Ec had lower LMR (*P* < 0.01) and RMR (*P* < 0.01) and higher SMR (*P* < 0.01), which showed a typical shade-avoidance and light exploitative strategy. However, the interaction between light and species was not significant for LMR (*F* = 1.40, *P* = 0.27), SMR (*F* = 1.75, *P* = 0.18), and RMR (*F* = 0.52, *P* = 0.67).

### 3.3. Leaf Morphology

**Figure 3:**
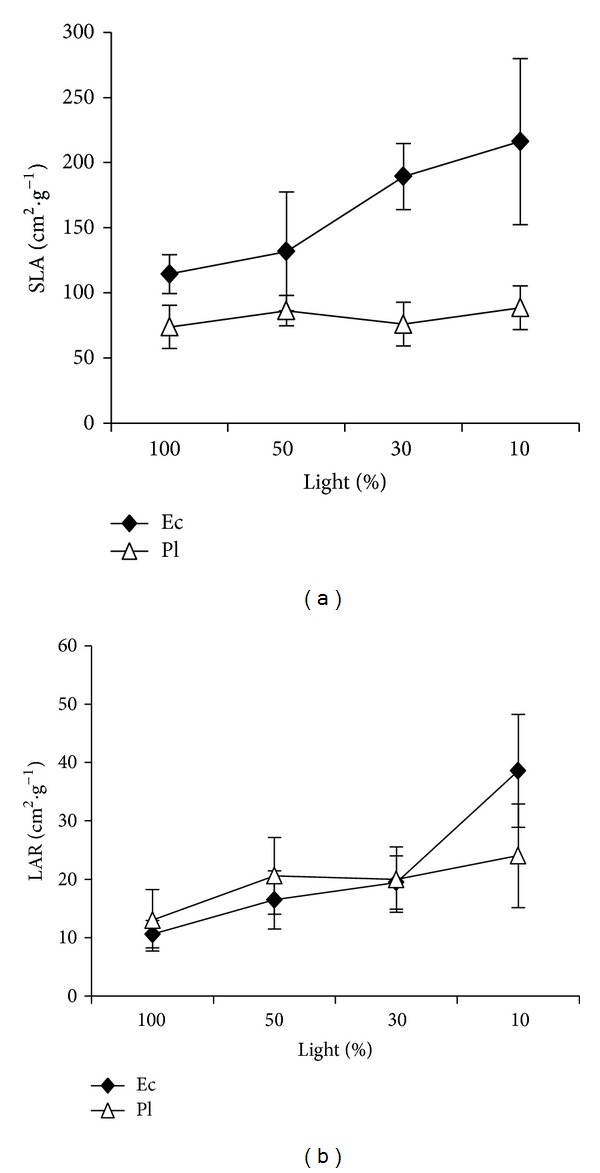
Leaf morphology responses to light for two rain forest tree species. Figures refer to (a) specific leaf area and (b) leaf area ratio.

There were significant differences for specific leaf area (SLA; *F* = 4.77; *P* < 0.01) and leaf area ratio (LAR; *F* = 13.17; *P* < 0.01) among the light levels. SLA and LAR increased with the decrease of light intensity (Figure 3), and they showed the highest values in 10% full light (*P* < 0.05). Ec showed higher SLA than Pl (*F* = 48.11; *P* < 0.01; Figure 3). However, Ec was similar to Pl in LAR (*F* = 0.72; *P* = 0.40; Figure 3). The interaction between light and species was significant for SLA (*F* = 3.67, *P* < 0.05) and LAR (*F* = 3.54, *P* < 0.05). With decreasing light intensity, Ec increased more rapidly than Pl in SLA and LAR.

### 3.4. Relationships between Growth Rate and Leaf Morphology

Morphological traits were strongly related to growth (Table 2). RGR was significantly positively related to SMR and SLA (except in 50% full light) and negatively correlated with LMR (except in 10% full light) under different light levels. Moreover, RGR was negatively correlated with RMR under 30% and 10% full light.

### 3.5. Morphological Plasticity

Leaf morphological plasticity was higher than biomass allocation plasticity (Table 3), which indicated that the change of organ forms was the major mode for plant species to adapt to different light environmental conditions. As a whole, Ec had higher morphological plasticity than Pl among measured traits.

## 4. Discussion 

The response of growth rate to environmental factors demonstrates different life-history strategies of tropical tree species [4, 27]. As the light intensity decreased, RGR of the two species decreased, suggesting they were light demanding but having different degrees of shade tolerance [22, 44]. Ec showed higher RGR and RGR_*H*_ than Pl. Higher growth rate provides a competitive advantage for species to escape quickly from the competition of surrounding species, especially higher height growth rate [45, 46]. So, Ec was more shade intolerant than Pl. Wood density was an important trait suggesting plant growth strategies [29]. Plant species with low wood density show higher growth rate and mortality rate in understory, while plant species with high wood density possess lower growth rate and higher survival rate [47, 48]. Air-dry density was 0.4 for Ec [49] and 0.6 for Pl [50], respectively. So, Pl invested more defense than Ec in order to increase its survival rate in understory. Higher growth rate and low wood density suggested that Ec was less shade tolerant than Pl.

Biomass allocation patterns to leaves, stems, and roots in plants demonstrate their adaptive strategies in different environmental conditions [51]. With the decrease of light intensity, the two species increased LMR, SLA, and LAR at the expense of roots in ways that contributed to the light interception ability [51, 52]. Ec had lower LMR and higher SLA, while Pl had higher LMR and lower SLA. Eventually, Ec was similar to Pl in LAR, indicating that there was no significant difference between them in light interception ability. Ec had higher SMR under each light level, indicating that it had an obviously shade-avoidance strategy [53]. Pl showed higher RMR, which was advantageous for them to escape carbohydrate lost, improve their resprouting ability, and increase survival rate in the understory [27]. However, RMR was negatively correlated with RGR. So, higher investment in root biomass for Pl did decrease its growth rate.

SLA is an important trait for plant to grow and survive in different light environments [54]. Ec had higher SLA, which was positively related to RGR in each light level. Higher SLA indicated higher photosynthetic capacity [19, 29, 55, 56]. Indeed, we did find that Ec had higher maximum net assimilation rate than Pl [57]. However, higher SLA did not favor Ec survival in low light due to low leaf defense ability. Salgado-Luarte and Gianoli [17] found that herbivory decreased the survival of the pioneer species* Aristotelia chilensis* (Elaeocarpaceae) in the forest understory but not in the canopy gaps. Pl had lower SLA than Ec, especially in 10% full light. Low SLA increased leaf defenses and leaf life span [12, 14–17, 23, 27], which was advantageous for Pl to survive in low light. Moreover, longer leaf life span would help plant to accumulate an extensive leaf area eventually and increase light interception ability in low light [23]. So, low SLA was an important regeneration manner for Pl.

Morphological plasticity is an important means by which plants cope with environmental heterogeneity. In this paper, plasticity of different organs was higher than biomass allocation, which was similar to the results of Bloor and Grubb [43]. The reason was perhaps that their adjustments could be achieved relatively cheaply [11, 43]. Ec showed higher morphological plasticity than Pl, which was in accordance with their shade tolerance degrees. And many researches also agreed that pioneer species had higher morphological plasticity than shade tolerant species [22, 25, 35]. Less plasticity for shade tolerant species to light is part of a general suite of traits associated with a conservative use of resources and a strong tolerance of low light stress [58]. Of course, some studies revealed that shade tolerant species had higher morphological plasticity [10, 36–38]. The conflict between shade tolerance and plasticity was perhaps caused by the selection of functional traits. So, how to select traits was important for us to compare the plasticity of different plant species.

The two tree species showed different adaptive strategies to light at different successional stages in the tropical montane forest on Hainan Island. Pioneer functional group, represented by Ec, showed higher light requirement. Higher photosynthetic capacity and biomass investment in stem favor them to grow faster and gain more light resources in high light environment. Moreover, high morphological plasticity favors them to acclimate to the dynamic light environments. However, low defensive ability due to higher SLA and lower RMR decreased their survival in low light. Late successional functional group, represented by Pl, demonstrated stronger defensive ability. Higher root allocation and tough leaves enhance their survivorship in the understory. Higher investment in root and leaf structure decreased the increment of leaf area and led to decreasing growth rate. Although the regeneration of Pl needed higher light intensity, longer life span after it reached the canopy made it exist in the old growth forest, such as long-lived* Eucryphia cordifolia* [59]. The above discussions on our study results suggest that a trade-off between growth and survival exists in tree species in the tropical montane rainforest of Hainan Island, and our results support the stress tolerance hypothesis.

The trade-off between growth and morphological traits means that early successional species increased their abilities of high growth to adapt to the high light environment through traits regulations, such as increasing SLA, SMR, and plasticity. However, this sort of traits regulations to increase growth rate could increase the risks of death such as animal herbivory, drought stress, and blowdown by typhoon, resulting in a lower survival rate. On the contrary, the climax species regulate their traits (such as decreasing SLA, while increasing RMR and LMR) so as to increase survival under the low light level understory at the expense of growth increment. The variations in the measured traits for the two species suggest that the tree species in this tropical montane rainforest have a trade-off in traits and growth rates under different levels of light intensity.

Differences in the ecological adaptive strategies among different functional groups at different stages provide the evidences for the maintenance of biological diversity in the tropical montane rain forest on Hainan Island. Since there are more than 700 tree species in this tropical montane rainforest, it is almost impossible to study the ecophysiological traits for each of them. The functional group approach is one of the ways to understand the ecology of tropical rainforest communities. There are varied ways for functional group identifications in tropical forests [60–65]. Species with similar morphological, phonological, or physiological traits can usually be categorized into the same functional group. In order to probe the characteristics (especially related with physiology) of the functional group, typical representative species from the group could be selected as the study subjects. The characteristics of the representatives can reflect the characteristics of the functional group. In this study, the two species were selected to represent the early and late successional stages of the tropical montane rainforest. They belong to the early successional functional group and late successional group, respectively. There are evidences (such as the morphological, distributional, and phonological similarities between the two representing species) that other species can be grouped into these two functional groups [61–65]. The species belonging to the early successional functional group include* Castanopsis fissa* (Fagaceae),* Schima superba* (Theaceae), and* Evodia glabrifolia* (Rutaceae), while those belonging to the late successional group include* Michelia mediocris* (Magnoliaceae),* Alseodaphne hainanensis* (Lauraceae), and* Beilschmiedia laevis* (Lauraceae).

## 5. Conclusion

Through traits regulations, pioneer and climax species could adapt to their own environment, making them occupy different sites or successional stages in the region, and different groups of species could partition their niche in both space and time. Consequently, the species with differing characteristics could coexist in the tropical montane rain forest region. The biodiversity of the forest region could be maintained.

## Figures and Tables

**Table 1:** Seedling traits, abbreviations, and units for growth and morphological analysis. These parameters were calculated following Poorter [42] and Bloor and Grubb [43].

Traits	Abbreviation	Units
Relative growth rate (ln⁡W_2_ − ln⁡W_1_)/(*T*_2_−*T*_1_)	RGR	mg g^−1^ d^−1^
Relative height growth rate (ln⁡*H*_2_ − ln⁡*H*_1_)/(*T*_2_ − *T*_1_)	RGR_*H*_	*μ*m *μ*m^−1^ d^−1^
Leaf mass rate (leaf dry mass/total seedling dry mass)	LMR	g g^−1^
Stem mass rate (stem + petiole mass)/total plant mass	SMR	g g^−1^
Root mass rate (root dry mass/total seedling dry mass)	RMR	g g^−1^
Specific leaf area (leaf area/leaf mass)	SLA	cm^2^ g^−1^
Leaf area ratio (total leaf area/total seedling dry mass)	LAR	cm^2^ g^−1^

*W* is seedling dry mass (g), *H* is seedling height (cm), *A* is seedling leaf area (cm^2^), and *T* is time (d). Subscripts refer to initial (1) or final (2) harvest.

**Table 2:** Correlations between growth rate and other measured variables in different light levels. Correlations significant at *P* < 0.05 are shown in bold.

Light intensity	LMR	SMR	RMR	SLA	LAR
100% full light	−**0.68**	**0.74**	−0.50	**0.67**	−0.37
50% full light	−**0.81**	**0.81**	−0.44	0.62	−0.38
30% full light	−**0.92**	**0.97**	−**0.74**	**0.89**	−0.22
10% full light	−0.63	**0.83**	−**0.72**	**0.86**	**0.72**

**Table 3:** Plasticity indexes for morphological parameters in seedlings of the three rain forest tree species under different light levels.

Indexes	Plasticity indexes		
	Ec	Pl	Mean
Biomass allocation			
LMR	0.49 ± 0.20	0.34 ± 0.03	0.42
SMR	0.14 ± 0.07	0.16 ± 0.06	0.15
RMR	0.30 ± 0.04	0.20 ± 0.03	0.25
Biomass allocation mean	0.31	0.23	0.27
Leaf morphology			
SLA	0.47 ± 0.12	0.17 ± 0.06	0.32
LAR	0.73 ± 0.13	0.46 ± 0.04	0.60
Leaf morphological mean	0.60	0.32	0.46
Total	**2.13**	**1.33**	
